# Knowledge mobilisation in practice: an evaluation of the Australian Prevention Partnership Centre

**DOI:** 10.1186/s12961-019-0496-0

**Published:** 2020-01-31

**Authors:** Abby Haynes, Samantha Rowbotham, Anne Grunseit, Erika Bohn-Goldbaum, Emma Slaytor, Andrew Wilson, Karen Lee, Seanna Davidson, Sonia Wutzke

**Affiliations:** 1 The Australian Prevention Partnership Centre, The Sax Institute, Building 10, 235 Jones Street, Ultimo, NSW 2007 Australia; 2 Menzies Centre for Health Policy, Charles Perkins Centre, The University of Sydney, Camperdown, NSW 2006 Australia; 3 Prevention Research Collaboration, Charles Perkins Centre, The University of Sydney, Sydney School of Public Health, Camperdown, NSW 2006 Australia

**Keywords:** Partnership research, knowledge mobilisation, co-production, evidence-informed policy, evaluation

## Abstract

**Background:**

Cross-sector collaborative partnerships are a vital strategy in efforts to strengthen research-informed policy and practice and may be particularly effective at addressing the complex problems associated with chronic disease prevention. However, there is still a limited understanding of how such partnerships are implemented in practice and how their implementation contributes to outcomes. This paper explores the operationalisation and outcomes of knowledge mobilisation strategies within the Australian Prevention Partnership Centre — a research collaboration between policy-makers, practitioners and researchers.

**Methods:**

The Centre’s programme model identifies six knowledge mobilisation strategies that are hypothesised to be essential for achieving its objectives. Using a mixed methods approach combining stakeholder interviews, surveys, participant feedback forms and routine process data over a 5-year period, we describe the structures, resources and activities used to operationalise these strategies and explore if and how they have contributed to proximal outcomes.

**Results:**

Results showed that Centre-produced research, resources, tools and methods were impacting policy formation and funding. Policy-makers reported using new practical methodologies that were helping them to design, implement, evaluate and obtain funding for scaled-up policies and programmes, and co-creating compelling prevention narratives. Some strategies were better implemented and more impactful than others in supporting these outcomes, with variation in who they worked for. The activities used to effect engagement, capacity-building and partnership formation were mostly generating positive results, but co-production could be enhanced by greater shared decision-making. Considerably more work is needed to successfully operationalise knowledge integration and adaptive learning.

**Conclusions:**

Describing how collaborative cross-sector research partnerships are operationalised in practice, and with what effects, can provide important insights into practical strategies for establishing and growing such partnerships and for maximising their contributions to policy. Findings suggest that the Centre has many strengths but could benefit from more inclusive and transparent governance and internal processes that facilitate dialogue about roles, expectations and co-production practices.

## Background

Knowledge mobilisation partnerships are increasingly recognised as a vital strategy in efforts to strengthen research-informed policy and practice [1–4]. These partnerships typically seek to combine the expertise of knowledge stakeholders across disciplines, sectors and jurisdictions (including policy-makers, practitioners, researchers, service users and communities) to improve the development, communication and implementation of evidence and innovations [5–7]. They have been found to increase the value of research by decision-maker partners; to enhance the policy and practice relevance of research outputs; to build intellectual capital (knowledge) and social capital (relationships) that strengthen the capacities of all parties to undertake, share and use research effectively; and to increase the uptake of research in policy and practice [3, 8–13]. It has been argued that the co-production of knowledge results in “*the best and most lasting influences of research*” [14] and has the potential to bring about systemic change [15].

Cross-sector knowledge mobilisation may be especially helpful for addressing ‘wicked problems’ such as chronic disease, where there are multiple, interconnected and contextually contingent causes, disputed and variable evidence, competing interests, and where solutions do not appear to be evident or fully attainable [2, 3, 16]. Collaboration can enable more effective responses to complex social problems than traditional research approaches [17] because it shifts the emphasis from ‘push’ and ‘pull’ models, where researchers disseminate findings or where decision-makers seek research, towards deliberative decision-making that blurs the distinction between those who produce knowledge and those who use it [18]. Cross-sector knowledge mobilisation can also aid navigation of the complex systems in which research is used, which are influenced by diverse institutional structures, disciplines, processes, priorities and discourses [19]. In such systems, expertise is distributed (and contested), interactions will be argumentative, political and values-orientated, implementation will probably require frontline ownership, and change will be emergent [20]. Thus, solutions may require people with diverse perspectives from different parts of the systems to work together as ‘active learners’ [21]. As Moss argues,

“*… knowledge mobilisation is not just about moving a clearly defined set of ideas, concepts, research techniques or information from here to there. Rather, it is about grappling with which forms of knowledge are apt in which contexts and how they can be strengthened through use*” [22].

The literature indicates that successful knowledge mobilisation partnerships have commonalities, including partners valuing different types of knowledge and contributions, participative processes in which the design, conduct, interpretation and implementation of research are negotiated and reciprocal, reflexivity and management of relationship dynamics, and an emphasis on building dialogue, trust, mutual respect and shared goals [21, 23–25]; however, this is not easy to achieve [26, 27]. Mobilising knowledge is a messy, conditional and profoundly context-dependent process [14, 28, 29]. Every partnership will have unique features and, like all complex systems, will be in flux [21], contingent on myriad factors, including relationships, values, leadership styles, incentives, structural and financial supports, role allocation, the type of problem being tackled, and their social and political contexts [3, 27, 30, 31].

Despite the increasing attention on knowledge mobilisation partnerships, we still know relatively little about how they are operationalised and enacted, i.e. precisely how they contribute to research-informed policy-making [32]. For example, the authors of a recent scoping review of 106 collaborative research partnerships were unable to identify relationships between the partnership’s activities and their outcomes due to insufficient detail about how the actual work was constituted. The authors asked that future collaborations report their implementation with enough detail so that any associations between structure, activities and outcomes can be identified [12]. This paper is a response to that request, using the Australian Prevention Partnership Centre as a case example.

### The Australian Prevention Partnership Centre

The Australian Prevention Partnership Centre (hereafter the ‘Prevention Centre’ or ‘Centre’) is a national collaboration established to undertake an integrated programme of research to improve the strategies and structures needed to prevent lifestyle-related chronic disease in Australia [33, 34].

The Centre strives to co-produce innovative, internationally significant research in systems science, economics, evaluation, implementation science and communication, including the development of new tools and methods for chronic disease prevention. It specifically targets entrenched complex problems where solutions are beyond the capacity of a single agency or field of expertise; more details are published elsewhere [33–36].

### Aims

This paper describes the knowledge mobilisation operations and proximal outcomes of the Prevention Centre. The aim is to provide a real-world case example of how a research collaboration is working in practice, and to share key learnings. We build on a previous paper that outlines the Centre’s knowledge mobilisation goals and the six strategies that were identified as crucial for achieving those goals [35]. Here, we describe the activities that have been used to operationalise those strategies and explore partners’ perceptions about whether and how these activities are contributing to outcomes.

## Methods

### Methodology

This paper uses data from the Centre’s ongoing evaluation. To better understand the relationship between elements of the Centre’s work and any observed effects, we drew on contribution analysis, which looks for plausible evidence that a strategy was implemented and that its theory of change was realised. The results provide a line of reasoning which may (or may not) indicate that the initiative contributed to the observed outcomes [37]. This approach has recently been adapted for assessing research impact [38], but our study takes a process evaluation approach in which we focus on the proximal indicators of collaboration such as engagement, capacity development, research production and decision-making processes as well as the early impacts of the Centre’s work on policy-making.

### The Partnership Centre programme model

**Fig. 1:**
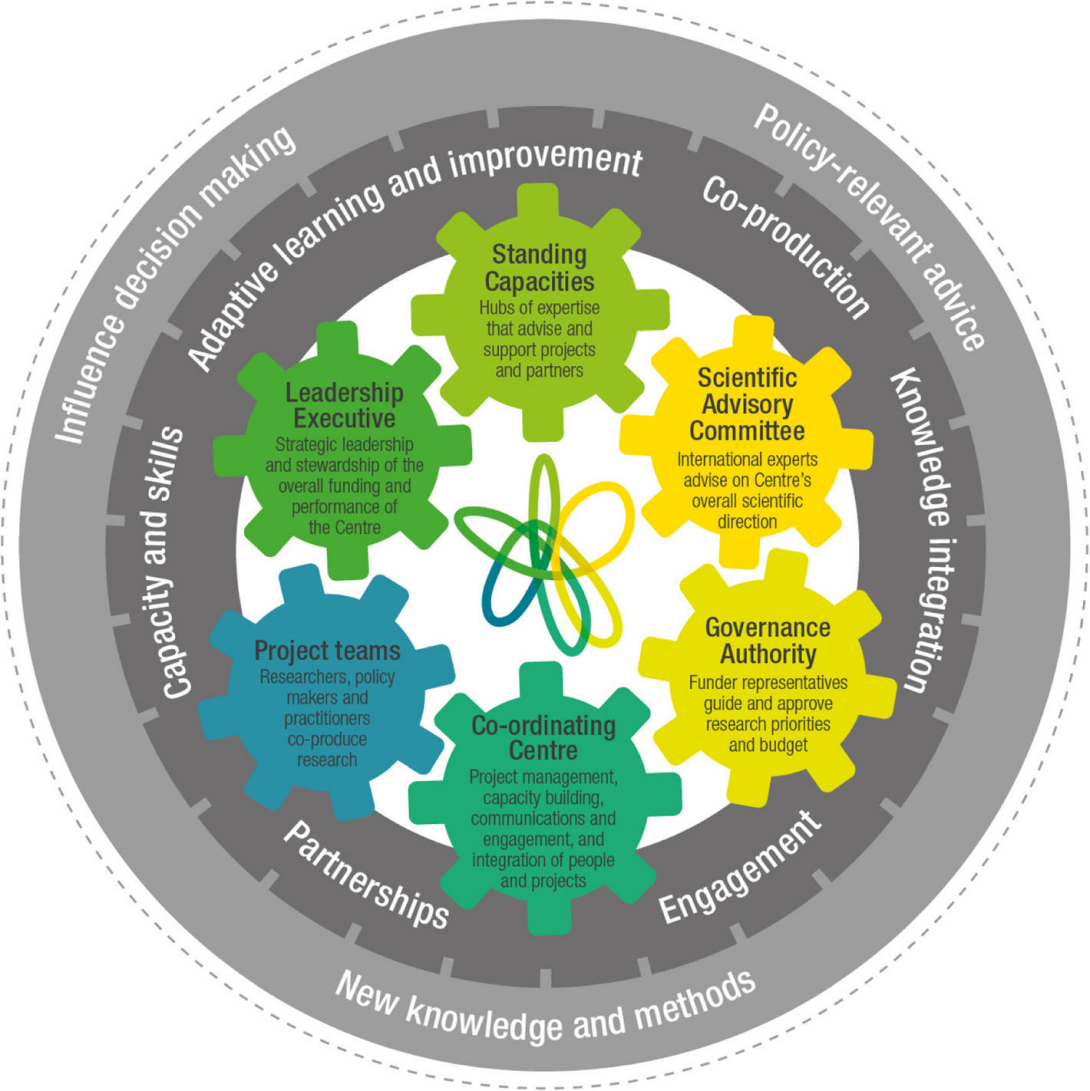
The Australian Prevention Partnership Centre model

A theory of change describes a desired pathway from activities to outcomes to impact. It unpacks the assumptions that lie behind an initiative and, where possible, backs these assumptions with evidence from research and stakeholder consultation [39]. This study uses the Prevention Centre’s programme model (Fig. 1) as its theory of change.

This model was developed in an earlier stage of evaluation using concepts from the literature and consultation with key stakeholders to articulate the in-practice functioning of the Centre [35]. It depicts the organisational structures and processes used by the Centre to facilitate collaboration (shown as cogs in the middle of the model), the six knowledge mobilisation strategies identified as necessary for achieving the Centre’s goals (shown in the inner circle), and the Centre’s objectives (in the outer circle). Additional files 1 and 2 provide more details on the components of the model. Here, we focus on the operationalisation of the six knowledge mobilisation strategies.

1. Partnerships

The Prevention Centre aims to forge strong intra- and inter-sectoral partnerships, founded on collegial relationships between individuals and formal agreement between organisations. This includes involving policy and practice partners in priority-setting, implementation planning and Centre governance (see Additional file 1 for a detailed overview of Prevention Centre governance and organisational structures).

2. Engagement

The Centre strives to build and maintain engagement by ensuring that members are aware of its activities, have access to useful resources and can make meaningful contributions to the Centre’s work. This includes funding teams of researchers, policy-makers and practitioners to work together on policy- and practice-focused research projects, hosting a range of interactive forums and resourcing a strategic communications team. See Additional file 5 for details of Prevention Centre events and Additional file 6 for an overview of its communication outputs.

3. Capacity and skills

The Centre aims to build a stronger prevention workforce by cultivating the capabilities of partners and the wider community to develop and use innovative fit-for-purpose research and tools, apply systems science and engage in cross-sector learning. This includes training for research and policy partners provided by experts in prevention and system change, and forums for project groups to come together on areas of common interest.

4. Co-production

The Centre seeks to ensure that partners genuinely work together to generate and use knowledge. In addition to the partnership-building activities outlined above, this includes promoting cross-sector investigator teams and establishing new research projects and collaborative opportunities in response to partners’ developing agendas.

5. Knowledge integration

Cross-fertilisation and integration of knowledge across projects and other sources is viewed as key to maximising the Centre’s impact. The aim is to support knowledge integration at three levels — micro (knowledge is shared and integrated within projects); meso (knowledge is shared and integrated across different but related projects and groups of investigators); and macro (knowledge from across the whole Centre is integrated and, where appropriate, combined with knowledge from other sources and sectors). Strategies include forums for partners to discuss and seek synergies across current and future projects and evidence synthesis.

6. Adaptive learning and improvement

Knowledge mobilisation is best facilitated by individuals and organisations engaged in adaptive learning and action [10, 40, 41]. This requires active information-seeking about ‘What is happening?’, ‘What is its significance?’ and ‘How we should act?’ [42]. Centre strategies include mixed-method evaluation activities and discussing responses to evaluation results in Centre forums.

### Data collection

A suite of evaluation activities has been undertaken in parallel with the Centre’s work, six elements of which informed this study, as shown in Table 1. Interview questions for chief investigators and funders were based on the Centre’s goals and so explored concepts relating to cross-sector partnership, co-production, innovation and skills development. The data from these early interviews is used in this study and informed the programme model described above (Fig. 1) [35]. This model, in turn, enabled us to refine subsequent interview questions and partnership survey questions. Participant feedback forms asked participants to comment on their experiences in relation to the learning objectives for each workshop. The interviews were conducted by three female researchers employed by the Prevention Centre (AH, SR & KL), who were experienced in qualitative data collection and analysis.

**Table 1:** Data collection for Prevention Centre evaluation

Method	Period	Objective	Data collection details	Analysis
1. Interviews with the Centre’s chief investigators and funding partners	January–March 2016	To explore experiences of involvement, perceptions about the Centre’s functioning and achievements, and areas for improvement	Semi-structured interviews with chief investigators (*n* = 21/31, including researchers and policy-makers) and funding partners (*n* = 5/5) named on the original grant; a total of 26 participants	Thematic analysis informed by research questions and guiding conceptual constructs on collaboration [43, 44]; NVivo 11 qualitative data management software [45] was used to support coding and analysis
2. Interviews with members of the Centre’s research network	July–August 2017		Semi-structured interviews with a representative purposive sample (selected by role and career stage) of PhD students, research officers/fellows and project leads, i.e. people involved in Centre research but not named on the original grant (*n* = 19); this was approximately 1/3 of the research network at that time	
3. Interviews with policy partners	June–July 2018		Semi-structured interviews with policy-makers (*n* = 18) who self-nominated for follow-up having completed a brief online survey about engagement with the Centre; the survey was advertised on the Centre website and via the Centre newsletter; one policy-maker was excluded from interviews because they had recently taken a paid role with the Centre All interviewees gave informed consent; interviews were audio recorded, professionally transcribed and then checked for errors by the interviewers	
4. Partnership survey (a cross-sectional anonymous online survey)	June 2015 October 2016 August 2018	To explore the Centre’s functioning according to partners (policy-makers, practitioners, researchers) and Centre staff; the survey covers perceptions of leadership, governance, resource allocation, collaboration and engagement	All Centre partners were invited to participate via personal email; survey hyperlinks were included in Centre e-newsletters and on its website; survey statements relating to aspects of the partnership were scored on a 7-point Likert scale from ‘strongly disagree’ to ‘strongly agree’; participants were also asked to rate specified experiences of partnership and comment on what worked well and what might be improved; the baseline survey was completed by 50 people, follow-up 1 was completed by 97 and follow-up 2 by 59 people	Statistical analysis of closed questions by wave of survey and thematic categorisation of open-ended questions; further details about the analysis of survey data are provided in Additional file 4
5. Participant feedback on ‘systems thinking’ workshops	Routinely collected after each event since February 2017	To elicit participants’ views of the functioning and value of events	Structured anonymous feedback forms completed by event attendees, including Centre partners and any other stakeholders who attended (*n* = 173 of approximately 230 attendees)	Descriptive statistical analysis and thematic categorisation of open-ended questions
6. Routine process data about Centre activities, funding and growth	Continual	To record Centre inputs, reach and outputs, including how strategies are being implemented and any impacts	Collation of data from project reports, communication products/website access data, project outputs, meeting minutes, the Centre’s partner database, ‘feedback register’ and key performance indicators	Thematic categorisation of text data and descriptive analysis of quantitative data

### Data analysis

We used a mixed methods approach to collate and synthesise this data. This took place at two time points, October–December 2017 and October–November 2018, and was performed by three of the authors (SR, KL and AH) who reviewed the existing data sources and coded data according to the knowledge mobilisation strategies identified within the programme model (Fig. 1) as well as inductively identifying additional themes. Survey data was analysed by EG and AG. Discussions between these researchers and other members of the evaluation team (SD, ES, SW) on interpretation of the findings increased the reliability of data analysis. To protect anonymity, only deidentified data was discussed within the wider author group. Analysis across the whole data set focused on building a picture of the Centre’s implementation and how stakeholders perceived its functioning and progress. See Additional file 3 for more detail about recruitment, data collection sources and analysis.

The overarching Prevention Centre evaluation was approved by the Sax Institute low-risk research assessment committee. Separate ethical approval for interviews with external stakeholders was given by the Sax Institute (Ref. R20180430).

## Results

Taking each of the Centre’s six knowledge mobilisation strategies in turn, we present a summary of how the Centre has tried to operationalise this strategy (i.e. we describe what has been implemented in practice) and give an overview of proximal outcomes. Table 2 provides an overview of the results, which are described in more detail below.

**Table 2:** Summary of results

Knowledge mobilisation strategies	Key governance and implementation strategies	Strengths and achievements	Stakeholders’ perceptions of benefits	Challenges and potential areas for improvement
1. Partnerships	• Involve partners in planning and governance • Require partners to commit resources so they have ‘skin in the game’ • Leverage existing cross-sector relationships to establish project teams, reach potential partners and create a networked platform • Connect with new partners and support current relationships	• Considerable growth in investigator team and partner organisations • Increased funding and resources from partners and government • Perception that skills are used effectively in the partnership and that the Centre’s benefits outweigh its costs	• Most interviewed policy-makers and funders regard the Centre’s work as useful, innovative and important • Policy-makers valued opportunities to shape research, access resources and forge connections within a collaborative network • Researchers valued linkage with (and more likely impact on) policy	• Partnership governance could be more transparent • Greater awareness of conflict resolution options needed • Some policy-makers found it hard to attend forums or to be ‘heard’ at them • Some uncertainty across stakeholders about how to tap into the Centre’s network
2. Engagement	• Funding teams of researchers, policy-makers and practitioners to work together • Interactive and networking forums for researchers, policy-makers and funders • Strategic communications, e.g. website, newsletters, narrative reports, policy/practice-friendly research summaries • Co-ordination and administrative support to link projects, manage funding and partnership agreements, and act as contacts for queries	• Partners see value in committing their time to the Centre and believe their abilities are being used effectively • Partners are getting the information needed to stay abreast of developments and opportunities, and to contribute meaningfully to the Centre • Most partners feel the Centre has a clear vision	• Access to high quality resources that are relevant and applicable to policy work • Awareness of Centre developments and opportunities • Engagement with systems science and other innovations • Access to online networked events and practice groups, and mentoring by Centre staff	• It has been hard to create a shared vision for all partners • Stakeholders can struggle to identify relevant projects or get involved in projects • Geographic distance from metropolitan areas and the coordination hub is a barrier • Belief that the partnership is achieving more than partners could do alone has decreased
3. Capacity and skills	• Dedicated capacity-building staff develop resources, run events and provide mentoring • Expert-run workshops and webinars • Cross-project forums and networks, including a community of practice in applied systems thinking • Investment in early-career researcher development (scholarships, postdoctoral fellowships and funding to attend conferences) • Cross-sector placements	• Capacity-building activities are frequent, varied, well-attended and well-received (e.g. perceived as useful and a good use of participants’ time) • High levels of reported satisfaction with the Centre’s communications, resources and capacity-building activities	• Access to national and international experts • Development and application of new knowledge and skills, e.g. in ‘real word’ research methods and systems approaches • Better understanding of the research-policy interface • Access to educational resources	• Cross-sector placements are hard to secure, often due to incompatible organisational requirements
4. Co-production	• Encourage cross-sector investigator project teams • Shape projects and collaborative opportunities around partners’ developing agendas • Host roundtable events and exchanges between researchers, policy-makers and practitioners to foster collective work and debate	• Multiple projects are engaged in cross-sector co-production • Many policy-makers are involved with different levels of seniority participating in different ways • Most policy-makers report examples of genuine co-production in which they saw themselves as full partners • Partners identify innovations arising from co-production	• Co-production allows partners to shape project directions (especially via shared priority-setting), gain access to expertise and resources, increase mutual learning and share ideas • Dramatically improved research relevance • Translation of research to policy is ‘built-in’ • Involvement in priority-setting justifies policy-makers’ time commitments	• Projects are less attuned to the needs of non-funding policy-makers as they are less involved in co-production • Different views of co-production: is it shared decision-making or generating research questions collectively or co-conducting research? • Greater facilitation of shared decision-making and problem-solving may be warranted • Co-production challenged by personalities, competing time frames and its own logistics
5. Knowledge integration	• Discussion forums to create linkages and synergies across current and future projects • Resourcing for high quality strategic evidence synthesis and communication • Dedicated roles and tasks regarding forging project connections, synthesising research findings and sharing knowledge	• To some extent, discussion forums are facilitating linkage and information-sharing	• In some cases, there are synergies across multiple projects	• More work is needed to create linkage, consolidate findings from separate projects and forge a coherent prevention narrative
6. Adaptive learning and improvement	• Evaluation: surveys, social network analyses, stakeholder interviews, process measures, key performance indicators and events feedback • Collate formal and incidental feedback in a register • Distribute evaluation results and discuss in Centre forums to ‘close the loop’ and enable action • Build reflection into the Centre’s quarterly reporting procedures	• There is some evidence of the Centre’s adaptivity and increasing flexibility	• In some cases, a dynamic and policy-responsive work plan	• More use could be made of evaluation information • Greater transparency at the executive level could help partners to see what information is considered and how it is acted on

To preserve anonymity, some of the illustrative quotes that follow have been modified (e.g. removing project titles or jurisdictional names), and two broad categories are used to identify the speakers: policy-makers and researchers; these are not mutually exclusive categories. For example, an individual member of the Centre may simultaneously work in a funding agency as a policy-maker, be a chief investigator for the Centre and have an adjunct academic appointment. Where their role is especially pertinent in contextualising or making sense of their views, we describe it in more detail. The term ‘partner’ is used to indicate policy-makers, practitioners and researchers who are formally involved in the Centre’s activities. The term ‘stakeholder’ is used more broadly to refer both to partners and anyone else who has registered for Centre events, resources or communications.

### Partnerships

Partnerships grew over the Centre’s initial 5-year lifespan. The investigator team grew from 31 to over 200 individuals based in 22 research institutions and additional practice settings. Partner organisations increased from 21 in 2014 to 36 in 2018, including academic and research institutions, government agencies and non-government/industry entities. Of the 40 research projects undertaken, 45% involve at least one non-academic investigator.

In-kind contributions from funding partners were initially estimated at $3.3 million across 5 years and were matched in real funding by Australia’s National Health and Medical Research Council (NHMRC). By the end of Year 5, this figure had almost doubled to $6 million, equating to $1.83 return for every $1 invested by the NHMRC. In-kind contributions included expertise from funding partners and academics, access to datasets and provision of office space. The value-proposition suggested by these increases was echoed in interviews where funders and other policy-makers talked about the work of the Prevention Centre as ‘important’, ‘relevant’, ‘useful’, ‘innovative’, ‘pragmatic’, ‘credible’ and ‘compelling’. As a funding policy partner explained,

“*In terms of in-kind* [contributions] *we’ve tripled what we thought would be given in that way because of the interest and the relevance of the work, so that’s something that’s been really important … The whole idea-sharing and the embedding research with embedded capacity-building has been a crucial component, and very positive.*”

Partnership survey data (Table 3) shows that perceptions of partnership governance strengthened in the first few years of the Centre, but scores dropped below 15-month follow-up levels at the 3-year follow-up point (this was a significant drop for items 7, 8 and 10). Management of conflict (item 9) had the lowest agreement at baseline but, at all timepoints, the majority of respondents fell into the ‘neutral’ category for this item, suggesting that they may have been unaware of a process for conflict resolution, possibly because they have not needed it. However, the data also indicated a perception that skills are used effectively within the partnership (item 3). Importantly, there was growth in agreement from baseline to the 3-year follow-up that the benefits of the Centre outweigh its costs (item 5).

**Table 3:** The Prevention Centre’s partnership survey results over three timepoints

Categories and statements in the partnership survey	Percentage agreement with survey statements		
	Baseline	15-month follow-up	3-year follow-up
Resource allocation			
1. Adequate financial resources are available	73.9	65.6	68.6
2. Necessary skills are available in the partnership	69.6	80.0	80.4
3. Available skills are used effectively	39.1	62.9	62.8
4. Adequate partner time is allocated	30.4	47.7	41.2
5. The benefits of allocating resources to the Centre outweigh the costs for my area	47.8	44.8	58.0
Governance			
6. There are defined roles and responsibilities	55.1	66.3	52.8
7. There is a clear process for planning and implementing activities	53.1	58.7	44.2
8. There is a clear process for shared decision-making	32.7	42.4	30.8
9. There is an effective process for managing conflict	10.2	29.7	9.6
10. There is a clear framework for monitoring progress	46.9	71.7	41.5
Leadership			
11. There is a clear vision for the Centre	46.0	63.9	64.4
12. There is clear communication of the goals of the Centre to staff	52.0	63.9	63.8
13. There is enthusiasm for achieving the Centre’s goals	68.0	82.5	81.4
14. There are strategies for relationship building among partners	54.0	74.2	61.0
15. There is strategic leadership for the Centre	60.0	81.3	76.3
Engagement			
16. I understand what the Centre is trying to achieve	71.1	79.6	67.9
17. I see value in committing my time to the Centre	84.4	82.8	75.5
18. I understand my role and responsibilities within the Centre	71.1	72.0	66.0
19. My abilities are used effectively in the Centre	40.0	57.0	54.7
20. I receive the information I need to contribute meaningfully to the Centre	48.9	66.7	58.5
21. I feel respected and valued as a member of the partnership	64.4	77.4	64.2
22. I believe the Centre partners are achieving more together than they could alone	55.6	81.7	69.8
Collaboration			
23. There is trust and respect among partners	65.9	79.3	76.9
24. There is sharing of ideas, resources and skills among partners	52.3	77.2	61.5
25. There is collaboration to solve problems	45.5	69.6	49.0
26. There is effective communication among partners	38.6	59.8	50.0
27. There are new and strengthened working relationships among partners	59.1	74.7	64.7

Policy interviewees identified several benefits arising from the Centre’s partnership activities, including having a voice and ability to shape research, access to expertise and resources, being part of a network that facilitated the sharing of ideas and generated synergistic dialogue, and further collaboration. Other interviewees indicated that the Centre was facilitating cross-sector relationships that would not have been developed in traditional programmes, but this seemed to favour formal partners who had a clear role in the Centre by virtue of their project position. New and strengthened connections were especially important as interviewees from both policy and academia frequently reported that their key motivation for involvement with the Centre was the opportunity it provides for research–policy partnership work.

Face-to-face forums had promoted informal networking and ‘built bridges’ between projects but there were difficulties using them strategically to foster debate and innovation, some partners struggled to find time to attend, and there was variable input by participants. For example, when project directions were outlined, this policy-maker did not feel able to voice his opinion,

“*… there were a couple of projects being discussed and the person I was sitting next to and I were going, ‘Hmm, I don't know how useful that's going to be’. But it’s not in the culture to say it in that kind of environment.*”

Many interviewees talked about the Centre’s network of research and policy experts as its major asset; however, a number of participants, especially more junior policy-makers and practitioners in non-funding agencies, indicated a desire for stronger ties to the network and expressed uncertainty about how to tap into it. This was echoed by some researchers who wanted greater cross-project collaboration but felt that being employed on discrete projects led by separate chief investigators created limited opportunities. Some were unsure if cross-project collaboration was permitted given they were paid from specific project budgets.

### Engagement

The partnership survey data (Table 3) indicated that perceptions of leadership and engagement increased in some areas from baseline to the first follow-up and remained higher than baseline at the 3-year follow-up (items 11, 19 and 20). The majority of participants across the time points indicated that they saw value in committing their time to the Centre (item 17). Participants’ belief that their abilities were being used effectively and that they were getting the information needed to contribute meaningfully to the Centre had significantly higher scores at both follow-ups compared with the baseline (items 19 and 20). The perception that the partnership is achieving more than partners could do alone increased significantly at the 15-month follow-up but decreased significantly at the 3-year follow-up to a level marginally higher than baseline (item 22). See Additional file 4 for a more detailed breakdown of data from the partnership survey.

Overall, interview data suggested that the Centre’s engagement strategy functioned well and contributed to outcomes but indicated some challenges during the Centre’s set up phase, including difficulties in developing a shared vision that embraced conceptual and pragmatic considerations and encompassed all partners. Some interviewees also mentioned opaque executive decision-making and governance processes, especially in relation to priority-setting and resource allocation,

“*… the actual communication of the decisions that are made and the processes could be clearer … how funding decisions are made, how priorities are decided on, what is the process if you've got an idea …* [do you] *first talk with decision-makers or do you need to talk to the management committee about it first?*” (Researcher)

Centre communications were identified as valuable in facilitating engagement by keeping stakeholders abreast of developments and opportunities, and by using techniques to maximise interest and impact,

“*In terms of the strengths of the* [Prevention Centre] *model, one has been maintaining communication with everyone. The newsletter that* [the Prevention Centre] *sends out is probably the only that I read ever because it is quite punchy, very useful and they only put points in there that would be relevant.*” (Researcher)

So, for many policy-makers, the policy-focus of the Centre’s communications and resources was key,

“*I often reach for things that the Prevention Centre has on their website … because they’ve been translated in very accessible language and also in a way that if we’re pitching something to the Minister, it says ‘Well what do we know?’ The ‘so what?’ is really helpful when you’re writing up something rapidly, to be able to see ‘Okay, what’s the practical implication of that?’ I find that very useful.*” (Policy-maker)

Some were excited by the Centre’s use of systems science and other innovations, and optimistic about where this would take them,

“*… we’re hopeful that’s going to bring about some change, but I think it’s already started bringing about some change in our own team … Whether or not we can then broaden that out to have an impact on others outside of the population health and planning area, remains to be seen. That’s definitely a work in progress but, certainly, the foundations are all there.*” (Policy-maker)

There appeared to be some patterns in engagement. Interviewees who reported having a clearly defined role within the Centre, especially if they received project funding, tended to feel more engaged; conversely, those who reported limited engagement often commented on a lack of projects that aligned with their area of expertise, or an unclear understanding of how they could be involved. Location and frequency of contact also seemed to make a difference; distance from major metropolitan areas (especially from Sydney, where the Centre’s coordinating team are based) and lack of regular interactions damped engagement, but participation in online networked events and practice groups, and mentoring by Centre staff, seemed to boost identification with the Centre and enthusiasm about its work.

### Capacity and skills

According to a range of data sources, capacity-building activities have been sufficiently frequent, varied, well-attended and well-received. In feedback forms, attendees generally stated that events were useful, a good use of their time and that they planned to attend subsequent events. For example, 80% of participants at systems thinking workshops who completed feedback forms said that they agreed or strongly agreed with the statement that ‘Insights generated through the workshop will help me better manage and apply a systems approach in my work’.

Across the evaluation data, partners reported that they valued the range of events and access to national and international experts, and had developed new knowledge and skills in areas including communications, research methods, and systems approaches,

“[The Centre] *is big on bringing internationally renowned speakers on systems thinking and other related areas to Australia. I always make a real effort to attend those because they're always such high quality. I'll research the speaker and look at their work and that leads you onto another body of literature that you hadn't considered.*” (Researcher)

They had also gained a better understanding of the research–policy interface and noted that cross-sector collaboration itself had built learning and capabilities, as this researcher noted,

“*By working with different professionals and people with different backgrounds, by default you’re building your capacity to understand the different domains and what their priorities are and how you might go about communicating something to one group vs another.*”

Learning was also facilitated by resources, such as factsheets and research summaries, which were accessibly written and available with minimal time lags. For policy-makers, information that ‘debunked myths’, included local data and used innovative approaches was especially welcome,

“*It’s information that is important, but it’s new information or it’s presented in a different way and I think this is part of that – the messages that we’ve had around for a long time aren’t quite working so we need new approaches, new research, new ways of telling the story. And I think that that’s what we’re really getting out of the Centre.*”

The investment in early career researchers was seen as key to developing the next generation of prevention researchers. However, despite a commitment to find placements for researchers in policy or practice agencies, and vice versa, these had been hard to secure, largely due to incompatible organisational requirements. Government agencies and research institutes tend to have security and access restrictions that would have excluded seconded staff from workplaces, data and/or technology. As an alternative, the Prevention Centre hosted research placements enabling policy staff and researchers to work jointly on projects. Feedback from these participants was overwhelmingly positive thanks to the level of support and opportunities provided.

The majority of policy interviewees said interaction with the Centre had advanced their knowledge of research, research projects, systems science and innovative work that was underway in other policy jurisdictions. They expressed enthusiasm about the Centre’s progress in developing practical methodologies that could help them design, scale up, implement, evaluate and obtain funding for policies and programmes. This included methods for community mapping, stakeholder engagement and interventions that are being piloted in different states. Research findings from Centre projects were also guiding policy agencies’ own research planning. Many said they were identifying risk factors, indicators, outcomes, measures or methods that were better equipped to tackle real-world conditions and valued the Centre’s work on co-developing compelling new prevention narratives.

Importantly, nearly all policy interviewees who were actively involved in Centre activities were able to identify examples in which ideas, information and/or data from the Centre had influenced policy and programme decision-making. These included the use of Centre data and modelling to inform advisory committees, agenda-setting, policy planning, investment strategies, programme implementation and scaling up, and monitoring and evaluation frameworks. For example,

“*The system dynamics modelling for childhood obesity has been a real success for us. It’s been incredibly beneficial for our programme planning and how we talk about the work that we’re doing, and what we expect the impacts to be.*”

Policy interviewees reported that their richest learning had been facilitated in three ways – first, by experiential application of tools and methods; second, by the availability of multiple learning and supportive opportunities which allowed them to select the best fit for themselves and/or to use opportunities complementarily; and third, through engaging in collaborative dialogue with researchers and with peers in other jurisdictions,

“*… some of it is around access to new knowledge and methods, particularly around systems thinking and approaches to improving prevention policy and practise, so that’s both an opportunity to learn in a structured way but also mentoring around some of those approaches.*”

The partnership survey asked respondents to rate their level of satisfaction with the Centre’s communications, online resources and capacity-building activities. Satisfaction levels had risen over the years, with between 73% and 92% saying, at the last survey, that they were satisfied/very satisfied with these functions (data not shown).

### Co-production

Co-production was highly valued because it allowed partners to articulate their needs and shape project directions, provided access to expertise and resources, ‘built-in’ the translation of research to policy, increased mutual learning and sharing of ideas, and dramatically improved the relevance of research,

“*It’s the participation which gets you thinking about your practice, gets you thinking about different* [policy] *opportunities. Because we’re involved in this work we don’t wait for some glossy two-pager because we co-created it, and we’ve communicated about it as we go.*” (Policy-maker)

However, this meant that some policy-makers outside of funding agencies who had less scope to co-produce research experienced the Centre’s outputs as less attuned to their needs.

Policy interviewees reported many examples of co-production in which they considered themselves full partners in key aspects of decision-making,

“[We are] *working collaboratively in co-production both with practitioners and researchers. That is often a motherhood statement … but I think the results do speak for themselves with the partnership in the Prevention Centre; that it really does work collaboratively and co-produce work.*”

Several senior policy-makers identified collaborative priority-setting as the crucial element in this process. Here, a policy-maker explained that she can invest time in Centre research because her manager knows their organisational priorities are being addressed,

“[The Centre] *goes through a prioritisation process with an executive which includes policy-makers which, I think, legitimises that and allows us — my colleagues and my staff — to legitimately work on projects and take a bit of time out to focus on it … because we’ve been actively involved in a co-creation process … and it’s doing work set through a priority-setting process — so, yeah, it’s more legitimate than other pieces of work.*”

Chief investigators, who had oversight of project teams, were particularly keen to advance a collaborative culture. They noted that co-production depended on the skills and experience of individual partners, bolstered by existing relationships, so that co-production was a familiar model for some teams but a cultural challenge in others. Researchers generally agreed that it was not possible to engage equally with all partners – personalities mattered (some policy-makers were less approachable or responsive) as did the quality of existing relationships.

Competing timelines were identified as a substantial challenge. Some researchers reported that government timelines had hampered projects or that policy partners had unrealistic expectations about how fast research outputs could be delivered. This was compounded by the time and logistics of co-production itself, as a chief investigator/researcher explained,

“*We’re going to lengths to work with people. We’re not making major decisions without the policy-makers there. We’re walking around finding out what research questions they are interested in, we’re feeding that back. We’re figuring things out as we go along which … means my projects are taking longer and I’m a bit of a pest, but it does take longer when you do it that way … it’s a pain in the arse because these are very senior policy-makers …* [and] *you literally can’t get them at two or three weeks’ notice. You have to get into their diaries a month in advance. So, getting all of the right people into the right room to have a meeting that reflects the equity of decision-making that you want takes time.*”

Overall, the data suggested some confusion about what co-production should look like and differences about the extent to which the Centre should adopt a ‘purist’ approach. For example, the first researcher quoted below argued that co-production means generating research questions collectively, while the second implied that policy partners should be co-conducting the research,

“*… true co-production is where the researchers and decision-makers sit together and come up with the research questions themselves. Whereas the way* [the Centre] *seems to work is the decision-makers have a question that needs to be answered or researchers think that there is something interesting and you take it to each other to see if you can actually make* [it fit] *… I don’t think there’s really ‘true co-production’ in terms of both researchers and decision-makers coming up with the questions collaboratively.*”

“*I can understand how it is that researchers end up doing most of the work in partnership research, they end up being the researcher, when in fact it is meant to be a little bit more equitable in the sharing of that role.*”

However, from policy-makers’ perspectives, co-production was not viewed as communal task completion but as shared decision-making, where policy-makers were actively involved in agenda-setting and ongoing deliberations, but not necessarily in the detail of the research (unless they wanted to be),

“*… they* [researchers] *are doing the heavy lifting of the analysis and doing a lot of the grunt work that we otherwise wouldn’t be able to do internally … So the strength to the Centre is that you’ve got someone there as a dedicated resource to focus on those issues.*”

There were many examples of policy-makers actively engaged in research project work but they tended to be both less senior and keen to develop a particular suite of skills. Senior policy managers generally wanted to minimise their time commitments while also playing to their strengths by focusing on strategic discussions where they had real power to shape the direction of projects. Unfortunately, they were not always offered this opportunity. Here, for example, a funding policy-maker explains the lack of opportunity to discuss research priorities,

“*I don't feel as though I am* [involved in priority-setting]*. No. I feel as though there’s a smorgasbord of opportunities that I can and cannot get involved in … So you nominate the ones that you have an interest in. I don’t think, others might disagree, that we had the opportunity to look at it as a whole and ask ‘Well, how does that contribute?’*”

These concerns were echoed by findings from the partnership survey (Table 3), which showed initial growth in partners’ positive experiences of collaboration (items 25 and 26 were significantly higher at the first follow-up) followed by a statistically significant decline, although all other items remained comparable to baseline. Less than half the respondents in the most recent survey agreed with the statement that ‘There is collaboration to solve problems’ in the Centre. These results may reflect the uneven process of collaboration seen in the qualitative data above but also, in the case of the Centre, the disorientation and uncertainty associated with the loss of a pivotal team member and coming to the end of a funding cycle.

### Knowledge integration

Research and policy interviewees both stressed the importance of knowledge integration for maximising the Centre’s impact. Discussion forums were perceived to support this goal, and a few policy partners gave examples of divergent projects being linked productively,

“*I was involved in some of the economic evaluation stuff. There were independent projects not really talking to each other, but* [a member of the leadership executive] *came in and then chaired all of them and ran a single meeting … that was fantastic because … there was a lot of overlap and then the projects were consolidated.*”

However, interviewees argued that more work was needed to consolidate findings from separate projects to provide “*the bigger picture in prevention*” and forge a coherent prevention narrative. As a chief investigator/policy-maker put it,

“*The thing that bothers me more is that I think it might look very scattered. We gave some money to this person and they did this and that was good and it got published. Somebody else did something else over here. I’m worried that it’s going to lack some kind of central organising theme that* [would make] *the Centre more than the sum of its parts.*”

### Adaptive learning and improvement

Adaptability was seen as crucial for implementing partnership research at this scale because projects must evolve in response to the needs of policy and practice partners, accommodate shifts in the political climate and provide space for innovative ideas to evolve. A few interviewees said they appreciated the Centre’s commitment to reflexivity and its willingness “*to ask hard questions of itself*”, and noted there was some evidence of adaptation, aided by the flexibility built into the original work plan,

“*I think it’s dynamic, and that’s great. I think it needs to be and it’s clearly being very responsive to emerging issues and to new ideas.*”

However, some interviewees (including investigators/funders, researchers and policy-makers) commented on the lack of transparency in decision-making at the executive level, which made it hard to determine what information was being considered and how it was acted on. It seemed the Centre had become more flexible as it had progressed, but that learning and improvement is sluggish in some areas. To take the example of shared decision-making that was signalled earlier, a few funders who were interviewed in 2016 raised concerns about their lack of involvement in making strategic decisions,

“*I think it’s been very mixed. I think we’ve helped develop the research questions but mostly the researchers are the ones who come forward with the questions. We've then had drafts to comment on but by the time you get draft as a proposal... It’s a bit late. You haven’t sat and brainstormed the research questions first together.*”

This view was supported at the time by a small number of chief investigators,

“*If someone was working in a true co-production mode, how would you know? What would you measure? In my mind, what you would measure is something around the integrated decision-making capacity. To what extent is there a shared responsibility for the project? Where are the decisions being made about what are the next steps and how things will be done, and what the data means? I actually don’t see a lot of that happening in the Centre.*”

This issue is also well established in the literature [25, 46] and, arguably, should be an axiomatic concern for a partnership committed to co-production. Yet, in the 2018 interviews with policy-makers, lack of shared decision-making was still a concern. Although some policy funders reported close involvement in specific key decisions, this was inconsistent, with limited capacity to set the direction of research programmes or, in some projects, even to be consulted in setting research questions or in developing the research plan,

“*… It was very tokenistic, and it wasn’t good because, essentially, at a planning session we were told that we were going to be involved in a project and then I hadn’t had any conversations with the person about the project. The first I heard about it was, I was asked to look at an ethics approval for the programme of research. And no one had spoken to us about any of the research. No one.”*

## Discussion

Evaluation data indicates that Prevention Centre stakeholders consider all six strategies in our theory of change (the inner ring of Fig. 1) to be important in building and sustaining an effective knowledge mobilisation partnership; this is not a surprising finding given that the model was itself developed when the Prevention Centre was already underway, drawing abductively on early evaluation in which partners played a central role.

However, the strategies appear to function variably, working better for some stakeholders than others. For example, partners were less likely to identify with the Centre when they received one-off project funding, and geographic distance and infrequent interactions appeared to have a negative impact on engagement. Additionally, not all partners felt involved in strategic decision-making, including funders who researchers would, presumably, want to keep ‘on side’.

Nevertheless, several important contributions were identified. Interviewees indicated that partnership and engagement strategies had contributed to them having a voice and ability to shape research, accessing research expertise and resources, being part of a network that facilitated the sharing of ideas and generated synergistic dialogue and further collaboration, and committing time and energy in Centre activities with the expectation of a return on investment. The capacity-building strategies seemed to be contributing strongly to engagement and there was considerable evidence from policy-makers that they had influenced some key outcomes, especially in relation to policy-makers’ uptake and application of research findings, methodologies and resources.

In many cases, the Centre’s approach to co-production was contributing to outcomes by increasing policy-makers’ engagement with and understanding of Centre research and ideas. With policy-makers’ involvement, the focus and outputs of projects were perceived as more policy relevant, pragmatic and likely to be used. However, some research questions were developed non-collaboratively or co-production fluctuated over the life of a project. This was a problem for policy-makers when they felt excluded from strategic decision-making processes, including opportunities to offer constructive critical input.

It was unclear if good or bad experiences of co-production were associated primarily with specific projects or individuals but there was evidence that they were shaped by structural and relational factors, including funding relationships, project timelines and personalities. Arguably, the funding model itself hampers co-production because tightly specified research plans have to be submitted with the funding application requiring the Centre to deliver predetermined projects that cannot respond flexibly to policy partners’ emergent priorities (and the frequent loss of these partners as they move to other jobs) when projects are operationalised years later. Researchers identified policy-makers’ availability and expectations as core barriers while policy-makers commented on researchers’ tendency to revert to ‘old school’ models of conducting research. This echoes co-production challenges reported by others, which include competing agendas and communication styles, epistemological conservatism by researchers limiting the scope for policy-makers to shape research priorities and methodologies, and the costs (time and money) of facilitating genuine partnership work [25, 46–49]. These challenges may have been exacerbated by partners’ different views about what co-production should look like, specifically, the tension between views that co-production is shared decision-making or generating research questions collectively or co-conducting research. The question of what constitutes co-production for different stakeholders would benefit from further research, including how a model of co-production can be developed that maximises pay-offs for all partners. In general, there is little guidance about establishing and managing research partnerships in health as well as an absence of criteria and case examples [50].

### Future directions

Findings suggest that the Centre’s governance structures, strategic leadership and internal communication processes, including ongoing dialogue about roles and expectations, could be strengthened. The literature on knowledge mobilisation partnerships emphasises the importance of participatory governance that formalises roles and supports connectivity and power-sharing [21, 25]. It also acknowledges the need to readjust governance responsively over the life-cycle of a partnership [51]. While interviewees generally reported positive experiences of the Centre, some comments echoed the survey data, which suggested concerns in relation to the Centre’s shared decision-making and collaboration. Policy interviewees saw themselves as contributors as much as consumers, and so were insistent that they should be engaged in developing (rather than simply receiving) research and ideas. Vacancies in key leadership, communications and capacity-building positions within the Centre may have contributed to slightly declining satisfaction in these areas, suggesting that support roles may benefit from review.

All interviewees recognised the importance of mutual understanding but there was a suggestion that researchers in particular may require improved ‘policy literacy’ [52], including greater recognition of policy-makers’ expertise. The challenge of working across epistemic cultures is well-recognised in the literature (e.g. [53, 54]) and previous studies indicate some enduring differences between policy-makers and researchers in how they view the same research/policy interactions. For example, Ellen et al. [55] found that policy-makers were more likely to emphasise contextual barriers to research use while researchers complained of communicative barriers. However, policy-makers may often understand research better than researchers understand policy [56]. Many interviewees claimed that they had learnt to work more effectively across the research–policy divide, but this was experiential learning that demanded active participation in partnership activities and, probably, some collaborative competency at the outset, so perhaps a greater emphasis on developing initial capacity for this work is warranted.

Key challenges in relation to knowledge integration were that projects are developed and funded discretely (and often mapped out years in advance), and hence there is a need to ‘retro-fit’ synergies, and the difficulty of integration in an organisation of the size, scope and necessary flexibility of the Centre. Some connections were forming between people and projects, but this seemed insufficient to meet the ambitious goal of consolidating knowledge synergistically to create a coherent prevention narrative. A dedicated Knowledge Mobilisation Fellow has now been appointed to lead the Centre’s knowledge integration work. Other suggestions included convening a steering group to explore how individual project outputs can contribute to a larger prevention story, a stronger emphasis on working towards clearly articulated shared goals, and developing theme groups to connect people with shared interests (although we also note that the Centre has already attempted the latter with little success, apparently due to different project phases and researchers viewing these linkages as ‘artificial’ with uncertain value).

As for adaptive learning and improvement, the Centre’s commitment to evaluation has resulted in a wealth of evaluation data. Some operations have evolved in response to feedback, but the information is not necessarily used responsively or well coordinated across activities and projects. Therefore, the Centre could benefit from closer consideration of (1) how to develop and embed adaptive operations and (2) what information is needed to guide adaptation. For example, we know that some partners do not understand or feel included in key decision-making but we have not gathered their ideas for how best to address this problem and remain uncertain about the root causes. The evaluation data indicates a mismatch between some researchers’ and policy-makers’ understanding of co-production, and this is echoed in the literature [46], but there may be additional or different factors at play. For example, are some projects less amenable to co-production? Do projects with more senior key players suffer because of poor availability? Are there structural or systemic problems such as the rigidity of project plans shaped by funding requirements, described above? Some argue that co-production is idealised (often hampered by interpersonal dynamics, competing values and conflicting interpretations of evidence)

and caution against its risks [27, 57]. Consequently, there are unlikely to be easy solutions to this specific problem, or to the larger issue of adaptive learning capacity, but both are pressing, especially now that the Centre is entering a new phase of expansion with more partners and projects, where participation and adaptive agility may be threatened by increasing organisational size and complexity. There is a rich literature on learning in professional and collaborative work which might give further guidance (e.g. [58–63]). This issue is especially important for the Centre given that reflexivity and adaption are necessary for innovation; without it, knowledge mobilisation partnerships can drift into “*translational ‘lock in’*” [64], privileging established models of research at the expense of creative ideas and methods that the Centre was founded to develop.

### Strengths and limitations

This paper has a limited focus on strategies and proximal outcomes relating to the Centre’s knowledge mobilisation model. It does not attempt to capture the many activities relating to the Centre’s individual research projects. Evaluative data about complex partnership research is dense, full of interdependencies and invariably entangles inputs, strategies and outputs [65]. Consequently, our results may be more reductive by comparison. However, we have tried to mitigate this by providing richer detail about the Centre’s structure and activities, and the conduct of this research, in the additional files.

Our sampling has important limitations. Interview participants volunteered to talk to a Centre researcher for up to an hour; this increases the likelihood that many interviewees were well-disposed to the Centre and, possibly, invested in it. The use of a Likert scale for most items in the partnership survey did not provide scope for respondents to state that they did not know if a service or process was available. The decline in scores in the most recent survey may indicate a need for the Centre to employ different strategies, but it could also signify a ceiling effect for some measures or be an artefact of the Centre’s difficult circumstances at this time (these include the tragic loss of the Deputy Director and a long period of uncertainty regarding on-going funding). Further, we do not know how representative the survey sample is of the overall partnership group at any time point. The findings, therefore, may not tell us the whole story and may not be applicable to the wider range of current partners. Finally, none of the evaluation data give us insights into the needs of potential stakeholders whom the Centre has not managed to engage. Future efforts could target members of the prevention community who currently have minimal or no involvement with the Centre to better understand their views and identify any barriers.

The Prevention Centre evaluation was conducted primarily for the purpose of learning and adaptation rather than for external accountability purposes. Given the internal improvement focus, the ‘insider’ approach taken is appropriate and provides opportunities to cross-check data against known factors in the Centre context [66]. We recognise the possibility that results can be skewed by the evaluators’ confirmation bias and/or interviewees giving socially desirable responses and we attempted to minimise this by prompting for frank critical feedback in interviews, guaranteeing anonymity to participants, triangulating data types and sources, and adopting a reflexive stance within the research team in which we discussed possible bias during analysis and writing up [67].

## Conclusions

Describing how knowledge mobilisation partnerships are operationalised in practice, and with what effects, can provide valuable insights for others considering entering into or forming such partnerships. Our results are not directly transferable – each partnership will have unique features and, like all complex systems, will be in flux [21], so strategies will work differently depending on local conditions, and a partnership’s antecedent structures, relationships and resourcing will shape what options are available in the first place. Yet, a rich description of activities and experiences across the Centre’s first 5 years, together with tentative theorising about how initial outcomes have been generated, should provide others with sufficient information to draw conclusions about the applicability of our findings in their contexts [68]. We also hope that being open about the challenges experienced by the Centre, and about our ongoing difficulties in traversing some of them, will contribute to wider dialogue about the value, demands and practicalities of collaborative knowledge mobilisation.

## Supplementary information

**Additional file 1.** Overview of Prevention Centre governance and organisational structures

**Additional file 2.** Knowledge mobilisation strategies: definitions, scope and objectives

**Additional file 3.** Evaluation data collection and analysis

**Additional file 4.** Change in agreement or satisfaction mean/median test for significance

**Additional file 5.** Overview of Prevention Centre events

**Additional file 6.** Overview of Prevention Centre communication outputs

## Data Availability

Some additional data generated or analysed during this study are included in supplementary information files. Interview data is not publicly available due to its identifiability. Data collection instruments are available from the corresponding author on request.

## Abbreviation

NHMRC: National Health and Medical Research Council
