# Supplementary material for: Knowledge mobilisation in practice: an evaluation of the Australian Prevention Partnership Centre
Source: Health Res Policy Syst. 2020 Jan 31;18:13. doi: 10.1186/s12961-019-0496-0 (PMC6995057; doi:10.1186/s12961-019-0496-0)
Supplement: Supplementary file 1 — Additional file 1. Overview of Prevention Centre governance and organisational structures [file 12961_2019_496_MOESM1_ESM.docx]

**Additional file 1. Overview of Prevention Centre governance and organisational structures**

| Governance Authority  The Governance Authority is a formal group convened to oversee the operations of the Prevention Centre and provide guidance relating to its management and activities. Members include representatives from funding partners and the Centre’s administering organisation who meet on a quarterly basis to:   - Review and provide advice on Centre work plans and projects, including monitoring progress and budgets - Monitor the overall performance of the Centre according to agreed indicators - Provide advice on the ongoing evaluation of the Centre - Provide advice on the Centre’s sustainability - Contribute perspectives on current policy matters that may impact the Centre |
| --- |
| Leadership Executive  The Leadership Executive is charged with overall leadership of the Centre, including performance management and implementation of the Centre’s work plan. It comprises eight members including the director and deputy director, standing capacity leads, selected chief investigators and a funding partner representative. They meet several times each year to:   - Provide oversight on operational, financial and administrative matters - Review and approve projects and Centre improvement activities - Review and progress the Centre’s work plan - Provide advice on the strategic direction of the Centre |
| Scientific Advisory Committee  This committee serves as an external reference for the Centre which links it to the international prevention, knowledge mobilisation and implementation research environment. It comprises four independent experts, two international and two national. Meetings are held bi-annually to:   - Provide oversight and advice in relation to the scientific validity, merit and integrity of the Centre’s work - Provide advice on scientific and strategic directions to ensure the Centre is meeting its objectives and is responding appropriately to and optimising changes in national and international prevention environments - Discuss ideas for future focus areas and how to further develop international partnerships |
| Coordinating Centre  The Coordinating Centre manages the Centre’s business including project initiation, funding and accountability processes, and delivering strategies to facilitate the partnership, including capacity building, internal and external communications and engagement, and integration of people, projects and learning. The Coordinating Centre consists of 5.4 full-time equivalent positions that fulfil roles including:   - *Leadership:* the Director and Deputy Director are responsible for leading the Centre’s strategic direction and decision making, as well as serving as a bridge between the operational elements of the Centre and Leadership Executive and Governance Authority. A large proportion of their roles focuses on building relationships, networking and facilitating engagement with internal and external stakeholders - *Administration*: the Associate Director, with support from administrative officers, is responsible for operations, processes and projects. This includes financial management, performance reporting and ensuring that projects are delivering against milestones - *Communications:* the communications team are responsible for developing and implementing activities to raise the profile of the Centre and to facilitate effective communication of evidence and innovations - *Learning and development:* this role lead efforts to understand the learning needs of members, developing and implementing capacity building activities as well as capturing the collective knowledge generated by the Centre. This position was replaced with a Knowledge Translation Fellow in March 2019. - *Evaluation*: two part-time fellows are responsible for evaluating and communicating the Centre’s lessons and challenges to drive continuous improvement and to contribute to knowledge about partnership research |
| Standing Capacities  Four standing capacities act as hubs of expertise that provide advice and support to projects and partners.   - *Systems Science and Implementation Capacity:* is led by one of the Centre’s Chief Investigators and comprises six academics across two universities, plus a manager of systems thinking and capacity building. The manager of systems thinking and capacity building builds individual skills and capacity for systems thinking across the Centre through developing accessible resources, delivering systems workshops, providing coaching and supporting individual projects as necessary, and facilitating a systems-focused community of practice. - *Rapid Response Evaluation Capacity:* is led by one of the Centre’s Chief Investigators and supported by a four-person research team. This capacity was intended to identify opportunities to embed research and evaluation in policy and programs, develop innovative methodologies to support high quality evaluations, provide advice, conduct evaluations nominated by funding partners and undertake analyses of population databases. Over time, this capacity expanded to include developing and implementing approaches for prevention, measurement and surveillance; undertaking evidence syntheses and providing training and support in complex evaluation methods. - *Synthesis Capacity:* This capacity is overseen by one of the Centre’s Chief Investigators, led by a Director and supported by a team of approximately eight research personnel. It was established to develop and apply novel methods for evidence synthesis and applies dynamic simulation modelling techniques to assist decision making. This technique brings together diverse evidence sources and uses a participatory approach to develop advanced computer models that can forecast the impact of various policy interventions. This provides policymakers with a unique ‘what if’ tool to test the likely impact of a range of possible solutions before implementing them. - *Communications Capacity*: This capacity was established to ensure that information about the Centre’s work and its outputs was readily available. It uses a website, and mainstream and social media, to communicate and to build community consensus about agreed prevention messages. Many of the communication activities attributed to this capacity were subsumed into the responsibility of the communications team as it expanded. |
| Project teams  The role of the project teams is to undertake the research projects specified with the Prevention Centre work plan. These teams are led by Chief Investigator(s) who have responsibility for leading project activities, supervising the research team and publishing findings. |
