# Supplementary material for: Knowledge mobilisation in practice: an evaluation of the Australian Prevention Partnership Centre
Source: Health Res Policy Syst. 2020 Jan 31;18:13. doi: 10.1186/s12961-019-0496-0 (PMC6995057; doi:10.1186/s12961-019-0496-0)
Supplement: Supplementary file 2 — Additional file 2. Knowledge mobilisation strategies: definitions, scope and objectives [file 12961_2019_496_MOESM2_ESM.docx]

**Additional file 2****. Knowledge mobilisation strategies: definitions, scope and objectives**

| **Engagement**  Within the Prevention Centre model, engagement is defined as committing time and resources to the work of the Centre and taking an active role in Centre activities (including undertaking research projects and capacity development, and attending meetings and events); feeling connected with the Centre, both in terms of knowing what is happening and having a sense of identity as a Centre partner; and sharing a common vision for the Centre and its goals. The Centre has a large and diverse network of partners working in a variety of settings (academia, policy, practice and industry) and geographically dispersed across Australia, all of whom have the potential to bring valuable knowledge, skills and experience that can enrich the work of Centre. |
| --- |
| **Partnerships**  Partnership refers to the formal and informal multi-disciplinary, intra- and inter-sectoral relationships between individuals, and to relationships between the Prevention Centre as an entity and other organisations and agencies (including universities, government agencies, and private companies). These partnerships involve negotiation and collaboration and operate for a variety of purposes, including undertaking research, building capacity and sharing knowledge and expertise. |
| **Capacity and skills**  This relates to the abilities of individuals, groups and organisations to produce and use evidence and innovations for improved chronic disease prevention. The Centre model recognises that (a) knowledge mobilisation in chronic disease requires that the prevention community (researchers, policymakers and practitioners) is able to work together to produce, access and use relevant knowledge, methods, and tools, and (b) that these individuals work at different levels of the systems with different levels of seniority (from early career to senior personnel and from those working on the ‘front-line’ to those involved in high level strategy and planning), and therefore have differing expertise and needs. Capacity and skills are thought to be enhanced through targeted capacity building activities and through participation in co-productive research processes. Efforts to develop knowledge and skills therefore emphasise: cross-sector collaboration; engagement in all stages of co-production; systems thinking tools and practices; understanding how to communicate evidence to a variety of audiences; and specialist expertise in key areas such as complex prevention program evaluation, health economics, evidence-based prevention practice and systems modelling. |
| **Co-production**  The Centre views co-production as *“collaborative knowledge generation by academics working alongside stakeholders from other sectors”* [[1](#_ENREF_1)]. Within the Centre’s model, co-production involves teams of policymakers and multidisciplinary researchers (and often practitioners and members of targeted communities) with diverse skills and perspectives working together on multiple aspects of a project. Co-production is a mechanism for knowledge mobilisation in its own right, but is hypothesised to be underpinned by other mechanisms including: ensuring local end-users have a voice in decision-making throughout the research agenda-setting and development process; having clear shared objectives; receptivity to and valuing of different kinds of knowledge and expertise; and integrating co-production into organisational structure and strategy. [[2](#_ENREF_2), [3](#_ENREF_3)] |
| **Knowledge integration**  It is increasingly recognised that policy- and practice-relevant evidence and innovation in health will come from multiple sources [[4](#_ENREF_4), [5](#_ENREF_5)], so knowledge integration refers to the process of synthesising and sharing knowledge from different yet complementary people, projects and organisations. The Prevention Centre conceptualises knowledge as much broader than research results [[6](#_ENREF_6)] so that it includes tacit experiential knowledge arising from practical experience and expertise within a particular domain (whether research, policy or practice) as well as formal explicit knowledge derived through systematic research and evidence gathering processes [[4](#_ENREF_4)]. Integration of knowledge across projects is viewed as key to ensuring that the Centre’s ‘whole is greater than the sum of its parts’. The Centre also recognises that many others are active in the prevention space so strives to forge connections and give partners what they need beyond the people and work being delivered by the Centre. |
| **Adaptive learning and improvement**  Adaptive learning and improvement is a process of continuous interaction between action, reflection, feedback and adaptation. It is an iterative cycle whereby information about interventions and their real-world implementation, together with judgements about their effectiveness, influence current and future activities. Developmental evaluation—which focuses on understanding initiatives while they are underway—is an appropriate way of formalising this process. Informed by systems thinking, it recognises that initiatives in complex settings are usually works-in-progress with key actors trialling strategies, tackling problems, renegotiating relationships and resources, and reviewing goals that emerge from unforeseen developments. The aim, therefore, is not to fine-tune a standardised initiative but to adapt so that projects and processes can be developed judiciously in response to changes in context, stakeholders, learning and ideas [[7](#_ENREF_7), [8](#_ENREF_8)]. Given the complex and dynamic nature of the Centre, which consists of many actors and components engaged in multiple projects and activities across research, policy and practice systems, the cycle of adaptive learning and improvement is hard to enact but crucial in enabling the centre to evolve productively. |
