# Supplementary material for: Knowledge mobilisation in practice: an evaluation of the Australian Prevention Partnership Centre
Source: Health Res Policy Syst. 2020 Jan 31;18:13. doi: 10.1186/s12961-019-0496-0 (PMC6995057; doi:10.1186/s12961-019-0496-0)
Supplement: Supplementary file 3 — Additional file 3. Evaluation data collection and analysis [file 12961_2019_496_MOESM3_ESM.docx]

**Additional file 3. Evaluation data collection and analysis**

| **Key informant interviews** | Key informant interviews were used to explore the progress and evolution of the Centre. Interviews were conducted either at the interviewee’s workplace or over the telephone. They were conducted with three key stakeholder groups:  Chief investigators and funding partners: Twenty one chief investigators (i.e. those academics named on the original funding application) and 5 representatives of the Centre’s funding agencies participated in key informant interviews between January and March 2016. A flexible topic guide was used to explore their experiences and perceptions of the Prevention Centre to date. Interviews were audio recorded and transcribed. Data was analysed using an inductive thematic analysis approach to generate data-driven themes across the data set.    Research network members: Researchers who are engaged in various roles across Centre projects include research officers/assistants, PhD students, and research fellows at different levels of seniority. This group is collectively referred to as the ‘Research Network’ and as at June 2017, consisted of approximately 55 members. Maximum variation sampling was used to purposively select a sample of 20 participants, 19 of who took part in an interview. A flexible topic guide was used to explore their experiences and perceptions of the Centre.  *Policymakers:* Interviewees were identified from an online survey of stakeholders based on in policy, program and services agencies (government, NGOs and health services delivery organisations) who had taken part in Prevention Centre workshops or events in the last three years (this was the most recent of three partnership surveys - see below). Seventy-nine people responded, and 53 respondents (67.1%) completed the whole survey [[9](#_ENREF_9)]. At the end of the survey respondents were asked if they would be willing to take part in an interview exploring their views and experiences and, if so, to provide their name and contact details. Twenty-two respondents indicated that they were willing to participate in interviews. One of these was deemed ineligible because she had subsequently left her government position and taken a paid role with the Prevention Centre. The remaining 21 were sent invitations to take part in an interview and 18 (86%) agreed.  Semi-structured interviews were conducted with these policymakers focusing on five domains: policymakers’ roles and context, their perceptions of the collaboration, what they were getting out of this approach (if anything), and their suggestions for the Centre’s future directions and improvement. Specific questions were asked about how the Centre’s model—including its goal to *“apply systems thinking to policy problems”*—was working in practice. Interviewees were encouraged to talk about their ‘real world’ experience and to define concepts in their own words.  Interviews with research network members and policymakers were audio recorded and transcribed. Fieldnotes were taken and used in analysis. Interview duration ranged from 27-63 minutes. Thematic analysis was also used for these interviews, but it included inductive identification of themes in the data and deductive searching for data relating to the dimensions of the program model which informed the interview questions. NVivo 11 qualitative data management software [[10](#_ENREF_10)] was used to support coding and analysis. Sampling adequacy was determined by coverage of the targeted participants, including the depth and range of views obtained in relation to our research questions, rather than assessment of theoretical or thematic saturation. [[11](#_ENREF_11)] |
| --- | --- |
| **Partnership Survey** | A prospective repeated anonymous survey study of Prevention Centre staff, partners, funding agencies and investigators. The survey was first conducted in June 2015 and follow-ups performed in September-October 2016 (15 month follow-up) and July-August 2018 (three year follow-up). The list of potential participants was generated from the Centre’s administrative contacts database and included chief investigators, policy and funding agency partners, project researchers and staff in the coordinating centre. Using this approach, personal emails were sent to 88 people for the baseline survey, and then 158 and 194 people, respectively. At each time point, a hyperlink to the survey was included in the Centre’s e-newsletter which was sent to everyone on the Centre’s mailing list, and was also available on the Centre’s website.  The 15 month follow-up response sample included baseline participants as well as staff who had joined the Partnership after baseline data was collected; the 3 year follow-up sample comprised 147 persons who had been invited to the 15 month survey and 47 persons new to the Partnership. No matched comparisons could be made as respondents participated anonymously at all time-points. Broad categories defined participants’ roles in the Centre but no other identifying information (such as age or sex) was collected. The survey has five key domains (leadership, governance, resource allocation, collaboration and evidence of partnership) each of which contains multiple statements. Each statement is scored on a 7-point Likert scale from “strongly disagree” to “strongly agree”. Participants are also asked to rate their overall experience of the five domains of partnership on a scale of 1 to 10, where 1 is an area that needs improvement and 10 is an area of strength.  The baseline survey ended with four open-ended questions to obtain more detailed feedback from participants about: 1) what is working well; 2) what needs improvement; 3) barriers and challenges; and 4) suggestions for improvement. In the follow-up surveys, participants were asked instead within each domain to comment on what worked well and how the domain might be improved and then to rate their satisfaction with aspects of the Centre’s operations. Finally, respondents were asked to provide any further comments.  Descriptive statistics were produced for individual questions (cross-tabulations) along with median and mean values. Baseline and follow-up responses on the change in means in individual questions were compared using linear regression across the three time-points, with multiple comparison for all pairwise (15 month vs baseline, 3 year vs baseline and 3 year vs 15 month) comparisons using the Tukey-Kramer adjustment; confirmation of results was performed using quantile regression (median) as the data were often negatively skewed. Where parametric and non-parametric results differ, the non-parametric are reported. All analyses were conducted using IBM SPSS Statistics 24 and Stata 15.1. |
| **Routine process data** | - The evaluation team collated and reviewed routine project and administrative data from across the Prevention Centre, including: - Communication products (newsletters, reports, factsheets) and website content - Event feedback surveys (anonymous) - Key Performance Indicators (reported on an annual basis to the NHMRC and covering a range of areas including expenditure, training, research outputs and event planning and organisation) - The Prevention Centre work plan - The Coordinating Centre Database, which contains details of Prevention Centre members, projects, and events - Quarterly project reports - Meeting Agendas, minutes and reports |
