# Supplementary material for: Knowledge mobilisation in practice: an evaluation of the Australian Prevention Partnership Centre
Source: Health Res Policy Syst. 2020 Jan 31;18:13. doi: 10.1186/s12961-019-0496-0 (PMC6995057; doi:10.1186/s12961-019-0496-0)
Supplement: Supplementary file 4 — Additional file 4. Change in agreement or satisfaction mean/median test for significance [file 12961_2019_496_MOESM4_ESM.docx]

Additional file 4. Change in agreement or satisfaction mean/median test for significance

|  | Baseline | | 15-month follow-up | | 3-year follow-up | | Comparison p-value | | |
| --- | --- | --- | --- | --- | --- | --- | --- | --- | --- |
| Statements | Mean | Median | Mean | Median | Mean | Median | *15 mo vs BL* | *3 yr vs BL* | *3 yr vs 15 mo* |
| Resource allocation |  |  |  |  |  |  |  |  |  |
| 1. Adequate financial resources are available | 5.6 | 6 | 5.5 | 6 | 5.6 | 6 | *0.923* | *0.968* | *0.779* |
| 1. Necessary skills are available in the partnership | 5.4 | 6 | 5.9 | 6 | 5.8 | 6 | *0.091* | *0.263* | *0.930* |
| 1. Available skills are used effectively | 4.8 | 5 | 5.4 | 6 | 5.4 | 6 | *0.025* | *0.040* | *0.992* |
| 1. Adequate partner time is allocated | 4.7 | 5 | 5 | 5 | 4.9 | 5 | *0.321* | *0.653* | *0.883* |
| 1. The benefits of allocating resources to the Centre outweigh the costs for my area | 5 | 5 | 5 | 5 | 5.3 | 6 | *0.999* | *0.525* | *0.453* |
| Governance |  |  |  |  |  |  |  |  |  |
| 1. There are defined roles and responsibilities | 5.1 | 6 | 5.5 | 6 | 5.1 | 6 | *0.130* | *0.991* | *0.157* |
| 1. There is a clear process for planning and implementing activities | 5 | 6 | 5.3 | 6 | 4.9 | 5 | *0.256* | *0.025** | *0.008** |
| 1. There is a clear process for shared decision making | 4.5 | 5 | 4.9 | 5 | 4.4 | 4.5 | *0.279* | *0.972* | *0.028** |
| 1. There is an effective process for managing conflict | 4.2 | 4 | 4.5 | 4 | 4 | 4 | *0.328* | *0.685* | *1.00** |
| 1. There is a clear framework for monitoring progress | 4.9 | 5 | 5.8 | 6 | 5.1 | 5 | *<0.001* | *0.717* | *0.007* |
|  |  |  |  |  |  |  |  |  |  |
| Leadership |  |  |  |  |  |  |  |  |  |
| 1. There is a clear vision for the Centre | 5.1 | 5 | 5.5 | 6 | 5.6 | 6 | *0.001** | *0.002** | *0.755* |
| 1. There is clear communication of the goals of the Centre to staff | 5.1 | 6 | 5.5 | 6 | 5.5 | 6 | *0.132* | *0.150* | *0.988* |
| 1. There is enthusiasm for achieving the Centre’s goals | 5.6 | 6 | 6 | 6 | 6.1 | 6 | *0.126* | *0.064* | *0.835* |
| 1. There are strategies for relationship building among partners | 5.3 | 6 | 5.9 | 6 | 5.5 | 6 | *1.00** | *0.532* | *0.144* |
| 1. There is strategic leadership for the Centre | 5.6 | 6 | 5.9 | 6 | 6 | 6 | *0.141* | *0.091* | *0.896* |
| Engagement |  |  |  |  |  |  |  |  |  |
| 1. I understand what the Centre is trying to achieve | 5.7 | 6 | 5.9 | 6 | 5.9 | 6 | *0.579* | *0.549* | *0.983* |
| 1. I see value in committing my time to the Centre | 6.1 | 6 | 6.2 | 6 | 6 | 6 | *0.900* | *0.878* | *0.557* |
| 1. I understand my role and responsibilities within the Centre | 5.7 | 6 | 5.8 | 6 | 5.6 | 6 | *0.879* | *0.942* | *0.643* |
| 1. My abilities are used effectively in the Centre | 4.8 | 5 | 5.4 | 6 | 5.1 | 6 | *0.009** | *0.023** | *0.470* |
| 1. I receive the information I need to contribute meaningfully to the Centre | 5 | 5 | 5.6 | 6 | 5.3 | 6 | *0.001** | *0.003** | *0.496* |
| 1. I feel respected and valued as a member of the partnership | 5.6 | 6 | 6 | 6 | 5.6 | 6 | *0.201* | *0.995* | *0.208* |
| 1. I believe the Centre partners are achieving more together than they could alone | 5.4 | 6 | 6.2 | 7 | 5.9 | 6 | *0.003* | *0.082* | *<0.001** |
| Collaboration |  |  |  |  |  |  |  |  |  |
| 1. There is trust and respect among partners | 5.5 | 6 | 5.8 | 6 | 5.9 | 6 | *0.173* | *0.114* | *0.887* |
| 1. There is sharing of ideas, resources and skills among partners | 5.2 | 6 | 5.7 | 6 | 5.6 | 6 | *1.00** | *0.157* | *0.949* |
| 1. There is collaboration to solve problems | 5.1 | 5 | 5.6 | 6 | 5.3 | 5 | *<0.001** | *0.531* | *<0.001** |
| 1. There is effective communication among partners | 4.8 | 5 | 5.4 | 6 | 5.2 | 5.5 | *0.001** | *0.375* | *<0.001** |
| 1. There are new and strengthened working relationships among partners | 5.3 | 6 | 5.8 | 6 | 5.7 | 6 | *0.112* | *0.360* | *0.868* |
| Overall rating (out of 10) |  |  |  |  |  |  |  |  |  |
| Leadership | 6.6 | 7 | 7.9 | 8 | 7.2 | 8 | *0.002* | *0.395* | *0.083* |
| Governance | 6.5 | 7 | 7.3 | 8 | 6.4 | 6.5 | *0.073* | *0.991* | *0.043* |
| Resource allocation | 6.6 | 7 | 7.4 | 8 | 6.7 | 7 | *0.041* | *0.984* | *0.052* |
| Collaboration | 6 | 6 | 7.4 | 8 | 6.9 | 7 | *<0.001* | *0.059* | *0.315* |
| Overall | 6.5 | 7 | 7.8 | 8 | 7.2 | 8 | *<0.001* | *0.078* | *0.132* |
| Total subscore (out of 35) |  |  |  |  |  |  |  |  |  |
| Leadership | 26.7 | 28 | 28.8 | 30 | 28.8 | 30 | *0.033* | *0.062* | *1.00* |
| Governance | 23.6 | 25 | 26 | 27 | 23.4 | 25 | *0.039* | *0.990* | *0.023* |
| Resource allocation | 25.5 | 27 | 26.3 | 27 | 27.1 | 28 | *0.654* | *0.294* | *0.689* |
| Collaboration | 25.9 | 28 | 28.2 | 30 | 27.7 | 28.5 | *0.034* | *0.194* | *0.817* |
| Personal experience (out of 49) | 32.6 | 35 | 41 | 42 | 39.5 | 41 | *<0.001* | *<0.001* | *0.431* |

* Non-parametric result used. Significance values are shown in bold.

Survey data analysis

Baseline and follow-up responses on the change in means in individual questions were compared using linear regression across the three time-points, with multiple comparisons for all pairwise (15-month vs baseline, 3-year vs baseline and 3-year vs 15-month) comparisons using the Tukey-Kramer adjustment; confirmation of results was performed using quantile regression (median) as the data were often negatively skewed. Where parametric and non-parametric results differ, the non-parametric are reported and marked with an asterisk. The subscales (totals for each domain) and overall ratings were reasonably normally distributed and so were analysed using linear regression only. All analyses were conducted using IBM SPSS Statistics 24 and Stata 15.1.
