# Supplementary material for: Knowledge mobilisation in practice: an evaluation of the Australian Prevention Partnership Centre
Source: Health Res Policy Syst. 2020 Jan 31;18:13. doi: 10.1186/s12961-019-0496-0 (PMC6995057; doi:10.1186/s12961-019-0496-0)
Supplement: Supplementary file 5 — Additional file 5. Overview of Prevention Centre events [file 12961_2019_496_MOESM5_ESM.docx]

**Additional file 5. Overview of Prevention Centre interactive activities (as of May 2019)**

| **Event type (number of events)** | **Purpose** | **Intended audience** | **Format** | **Topics covered included** | **Dates (events per year)** | **Average attendees (range)** | **Location (events per location)** |
| --- | --- | --- | --- | --- | --- | --- | --- |
| Investigators Forum (10) | To encourage knowledge sharing across projects and discuss strategic direction for the Centre | Internal: Prevention Centre Chief Investigators and project staff | Face to face: One-day sessions usually consisting of presentations and group discussions. | Strategic planning  Reflections on progress  Project updates and discussions | 2013 (2)  2014 (1)  2015 (2)  2016 (2)  2017 (2)  2018 (1) | 37 (30-58) | Sydney (8)  Melbourne (2) |
| Research Network Forums (8) | Provide opportunities for research staff to network, hear about each other’s work, and gain new skills | Internal: Prevention Centre project staff, including research officers, PhD students and early to mid career researchers. | Face to face. One-day sessions usually consisting of presentations and/or panel sessions and group discussions. | Project updates  Speed networking  Capacity building in areas such as knowledge to action, working in partnership and knowledge co-production, communication skills, and systems approaches  Insights into policy (including a day in the life’ at NSW Health, stories of policy influence) | 2014 (1)  2015 (3)  2016 (2)  2017 (1)  2018 (1) | 24 (13-42) | Sydney (6)  Melbourne (2) |
| Workshops, Roundtables, Masterclasses (46) | Share knowledge and expertise, build the profile of the Centre and provide networking opportunities | Event dependent but may include Prevention Centre chief investigators, project staff, and policy and practice partners, as well as external stakeholders from academic, policy and practice settings | Face to Face. Usually half- or full-day sessions consisting of presentations and group discussions. | Think2impact  Systems approaches  Workplace insights  Partnerships in Public Health  Health economics  Critical Appraisal (Synthesis Capacity)  Dynamic simulation modelling (Synthesis Capacity)  Complex Program Evaluation (Rapid Response Evaluation Capacity)  Implementation and scale up  Systems approaches to prevention (Systems Science and Implementation Capacity) | 2014 (8)  2015 (12)  2016 (5)  2017 (7)  2018 (14) | 23 (5-69) | Sydney (27)  Melbourne (10)  ACT (6)  South Australia (1)  Tasmania (1)  Northern Territory (1) |
| Online course (2) | To develop expertise in evaluating complex programs evaluation | Internal and external stakeholders in academic, policy and practice settings | Online course for five weeks | Complex Program Evaluation | 2017 | 59 | Online (2) |
| Public lectures (10) | To share knowledge and expertise, build the profile of the Centre and provide networking opportunities | Academics and policymakers (inside and outside of Prevention Centre) | Face to face presentations by national and international experts on topics of relevance to chronic disease prevention | Public Health Law (Scott Burris)  Food industry (Diane Finegood)  Linking research, policy and practice in a complex world (Allen Best)  Translating systems thinking into public health innovations (Terry Huang)  Nudge theory and public health (Mike Kelly)  Systems approaches for physical activity and obesity (Harry Rutter) | 2014 (2)  2015 (3)  2016 (3)  2017 (1)  2018 (1) | 60 (25-160) | Sydney (10) |
| Webinars (5) | To share knowledge and expertise, build the profile of the Centre | Internal and external stakeholders in academic, policy and practice settings | Online presentations by experts from within and outside Prevention Centre on topics of relevance to chronic disease prevention | Evaluating new international classifications of public health interventions (Nicola Fortune)  Mobilising knowledge in complex systems (Diane Finegood and Bev Holmes)  Best practice in obesity policy (Gary Sacks)  Framework for systems change (Penny Foster-Fishman)  A healthy food intervention model (Dylan Knowles) | 2016 (2)  2017 (2)  2018 (1) | 135 (27-280) | Online (5) |
