# Supplementary material for: Knowledge mobilisation in practice: an evaluation of the Australian Prevention Partnership Centre
Source: Health Res Policy Syst. 2020 Jan 31;18:13. doi: 10.1186/s12961-019-0496-0 (PMC6995057; doi:10.1186/s12961-019-0496-0)
Supplement: Supplementary file 6 — Additional file 6. Overview of Prevention Centre communication outputs [file 12961_2019_496_MOESM6_ESM.docx]

**Additional file 6. Overview of Prevention Centre communication outputs (as of May 2019)**

| **Output/activity** | **Brief description** |
| --- | --- |
| Fortnightly update | Succinct emails sent to internal stakeholders, which include information on projects, learning and development opportunities, events, staff updates and other relevant news. |
| Investigator teleconferences | Regular teleconferences for members of the Chief Investigator and Research Network groups in addition to face to face meetings provide opportunities for members to be updated on what is happening across the Prevention Centre and to discuss their current projects and findings. These teleconferences occur two to three times a year, usually in between the face to face meetings. |
| ‘The Chronicle’ newsletter | ‘The Chronicle’ is a bi-monthly newsletter sent to over 2,000 subscribers that provides up to date information on progress of ongoing projects, results and impacts of completed projects, upcoming conferences and events, policy matters and announcements impacting on the prevention of chronic disease. |
| Evidence Reviews | Reviews (usually rapid reviews) of evidence designed to identify, synthesise and interpret the latest evidence and provide a concise summary of the current state of knowledge. |
| Evidence Briefs | Evidence briefs are concise summaries of key findings of Evidence Reviews, aiming to summarise the evidence in a form that can be quickly digested by policymakers. |
| Findings Briefs | These summarise key findings and outcomes of each completed research project, using infographics to convey information succinctly for policy makers. |
| Prevention Factsheets | These are factsheets on key and current issues in prevention, which aim to distil evidence in a clear and concise manner and provide evidence-based “sound bites” to help policymakers argue for action and investment in prevention. |
| Impact case studies | Short case studies illustrating areas in which the Prevention Centre research has influenced policy or practice. |
| ‘In a nutshell’ articles | Short Q&A article explaining key findings and importance of published journal articles based on Prevention Centre research. |
| Progress Report | Periodic report-style publication that conveys key achievements, findings and impact of the Prevention Centre. |
| Podcasts | Our podcast series consists of interviews with Prevention Centre researchers on general topics around prevention of chronic disease, aimed at a broader public health audience and the general public. |

The resources listed above are available on the Prevention Centre’s website <https://preventioncentre.org.au>. Users frequently visit the website with 457,666 page views and 218,587 users (those who have initiated at least one session) since the website’s launch in March 2015.

**Additional file references**

1. Greenhalgh, T., et al., *Achieving Research Impact Through Co‐creation in Community‐Based Health Services: Literature Review and Case Study.* The Milbank Quarterly, 2016. **94**(2): p. 392-429.

2. Heaton, J., J. Day, and N. Britten, *Collaborative research and the co-production of knowledge for practice: an illustrative case study.* Implement Sci, 2016. **11**: p. 20.

3. Holmes, B.J., et al., *Mobilising knowledge in complex health systems: a call to action.* Evidence and Policy, 2016.

4. Glasgow, R. and K. Emmons, *How can we increase translation of research into practice? Types of evidence needed.* Annual Review of Public Health, 2007. **28**: p. 413–433.

5. Green, L., *From research to “best practices” in other settings and populations.* American Journal of Health Behavior, 2001. **25**(3): p. 165–178

6. Best, A. and B. Holmes, *Systems thinking, knowledge and action: towards better models and methods.* Evidence & Policy: A Journal of Research, Debate and Practice, 2010. **6**(2): p. 145-159.

7. Patton, M.Q., *Developmental evaluation: applying complexity concepts to enhance innovation and use*. 2011, New York: Guilford Press.

8. Patton, M.Q., K. McKegg, and N. Wehipeihana, *Developmental evaluation exemplars: Principles in practice*. 2015, New York: Guilford Publications.

9. Rowbotham, S. and H. Hughes, *Draft impact report (26 March)*. 2018, The Australian Prevention Partnership Centre: Unpublished.

10. QSR International Pty Ltd, *NVivo qualitative data analysis software. Version 11*. 2017.

11. O’Reilly, M. and N. Parker, *‘Unsatisfactory Saturation’: a critical exploration of the notion of saturated sample sizes in qualitative research.* Qualitative research, 2013. **13**(2): p. 190-197.
